# Evaluating rectal swab collection method for gut microbiome analysis in the common marmoset (*Callithrix jacchus*)

**DOI:** 10.1371/journal.pone.0224950

**Published:** 2019-11-07

**Authors:** Stephen C. Artim, Alexander Sheh, Monika A. Burns, James G. Fox

**Affiliations:** Division of Comparative Medicine, Massachusetts Institute of Technology, Cambridge, Massachusetts, United States of America; Tulane National Primate Research Center, UNITED STATES

## Abstract

The common marmoset (*Callithrix jacchus*) is increasingly used as an animal model for biomedical research; however, gastrointestinal diseases causing significant morbidity are endemic in many captive marmoset colonies. Establishing gut microbiome patterns in a marmoset colony may aid in clinical decision-making and model reproducibility. A standardized method of sample collection and storage is essential for proper interpretation of microbiome data. While microbiome studies commonly utilize fecal samples, the goal of this study was to determine whether the microbiome profile from a rectal swab performed on a sedated animal was comparable to the microbiome profile from a fecal sample. During routine physical exams, paired fecal and rectal swab samples were collected from each of 23 marmosets. DNA was extracted from all fecal and rectal swab samples and 16S ribosomal RNA gene sequences were amplified and analyzed. Initial comparison of the relative abundance of bacterial phyla between paired samples had a r^2^ value of 0.70 with S of 0.08 with no significant differences in α and β diversity metrics between fecal and rectal samples. Initial analysis however, revealed 5 discordant fecal-rectal pairs which corresponded only with the 5 rectal swabs that were classified as free of visible fecal matter during collection. Exclusion of these 5 pairs resulted in an optimized fit of the data as evidenced by a r^2^ value of 0.91 with S of 0.05. These results demonstrate that rectal swabs are a reliable method for profiling the fecal microbiome in the marmoset since the bacterial composition from a rectal swab with visible fecal contents correlated well with the bacterial composition from a fecal sample from the same marmoset. This study highlights the importance of standardized sample collection methods and exclusion of inappropriate samples.

## Introduction

The common marmoset (*Callithrix jacchus*) is a New World primate that is an attractive nonhuman primate animal model in biomedical research due to its small size, reproductive traits, low zoonotic risk as well as genetic and physiologic similarity to humans. The common marmoset has been used in neuroscience, behavioral, toxicological, reproductive biology, stem cell biology, transgenic, obesity, autoimmune and infectious disease studies[1,2]. Historically, the average life span of captive marmosets has been reported to be 5–7 years[3]. However, other studies documented longer average lifespans of 9–12 years, but the reason for this disparity is unclear[4–6]. Colony dynamics could impact average lifespan, since one colony that reported longer average lifespans also observed fewer incidences of gastrointestinal (GI) disease[5]. GI disease has been shown to be one of the primary causes of death in captive marmosets less than 6 years of age in conventional colonies[3,6]. GI disease, including inflammatory bowel disease, previously referred to as marmoset wasting syndrome is endemic in many captive colonies of marmosets[7]. Understanding the etiology of chronic conditions that influence the average life span and health of an animal is essential for improving the health of these valuable animal models of human disease.

Studies in humans and other laboratory species have shown that a disturbance in the gut microbiota, known as dysbiosis, is associated with a wide spectrum of diseases[8]. Documenting and understanding differences in the gut microbiome between health and disease states could eventually lead to insight on the etiology and pathogenesis of the disease, identify targets to serve as biomarkers for initial disease presentation, or potentially lead to therapeutics for the disease[9]. Surprisingly, there is limited information regarding the species-specific composition of the gut microbiota in common marmosets. A recent study that compared survival and causes of morbidity in specific pathogen free (SPF) barrier-raised common marmosets with conventionally-housed common marmosets reported greater survival rates and no deaths associated with GI disease related to intestinal infections in the SPF barrier colony[10]. The study reported significant differences in the gut microbiome of common marmosets in the conventional colony compared to that of the SPF barrier colony[10].

Previous microbiome studies have demonstrated the importance of standardized techniques for sample collection, storage, DNA extraction, and library preparation[11–14]. The goal is to utilize techniques that minimize DNA alterations in the samples which would affect the analysis and results. The current gold standard is to freeze the sample immediately[11]. Studies in humans have shown that the composition of the microbiota in fecal samples frozen and stored to 6 months at -80°C are not significantly different than using fresh samples[12,13].

Since marmosets do not always defecate when moved to a clean cage, collecting contaminant-free fecal samples reliably from marmoset presents a challenge. Furthermore, due to the high rates of social housing of marmosets with conspecifics, there is an increased chance of cross contamination of fecal samples from cagemates if feces are collected from the cage pan.

Another important consideration in developing standardized sampling protocols is obtaining samples that are informative and reliable about the individual being sampled. There is increased awareness of differences in the microbiome from diverse sections of the GI tract. McKenna et al. reported that the microbiome of fecal samples is not representative of the microbiome of the colon from healthy and sick rhesus macaques (*Macaca mulatta*)[15]. In contrast, a more recent study using all healthy rhesus macaques showed that the fecal microbiome is representative of the colonic microbiome[16]. A human study demonstrated that the microbiota from feces and a rectal swab sample from the same person were highly similar[11].

Establishing how well the microbiome of a rectal swab represents the microbiome of a fecal sample is important for interpreting future lower bowel microbiome studies in the common marmoset. As part of routine physical examinations, marmosets within our colony are sedated for sample collection and examination. This presented a unique opportunity to collect rectal swabs reliably via a simple and convenient method in a sedated marmoset. In this study, we hypothesized that there would be no significant difference in the microbiome profiles from fecal samples compared to samples collected via a rectal swab in a cohort of common marmosets. To test this hypothesis paired rectal and fecal swab samples from 23 marmosets were collected, and the 16S ribosomal RNA gene sequences were amplified and analyzed to determine the similarity of microbiome profiles for each fecal-rectal pair.

## Results

### Location of rectal swab sampling

**Fig 1:**
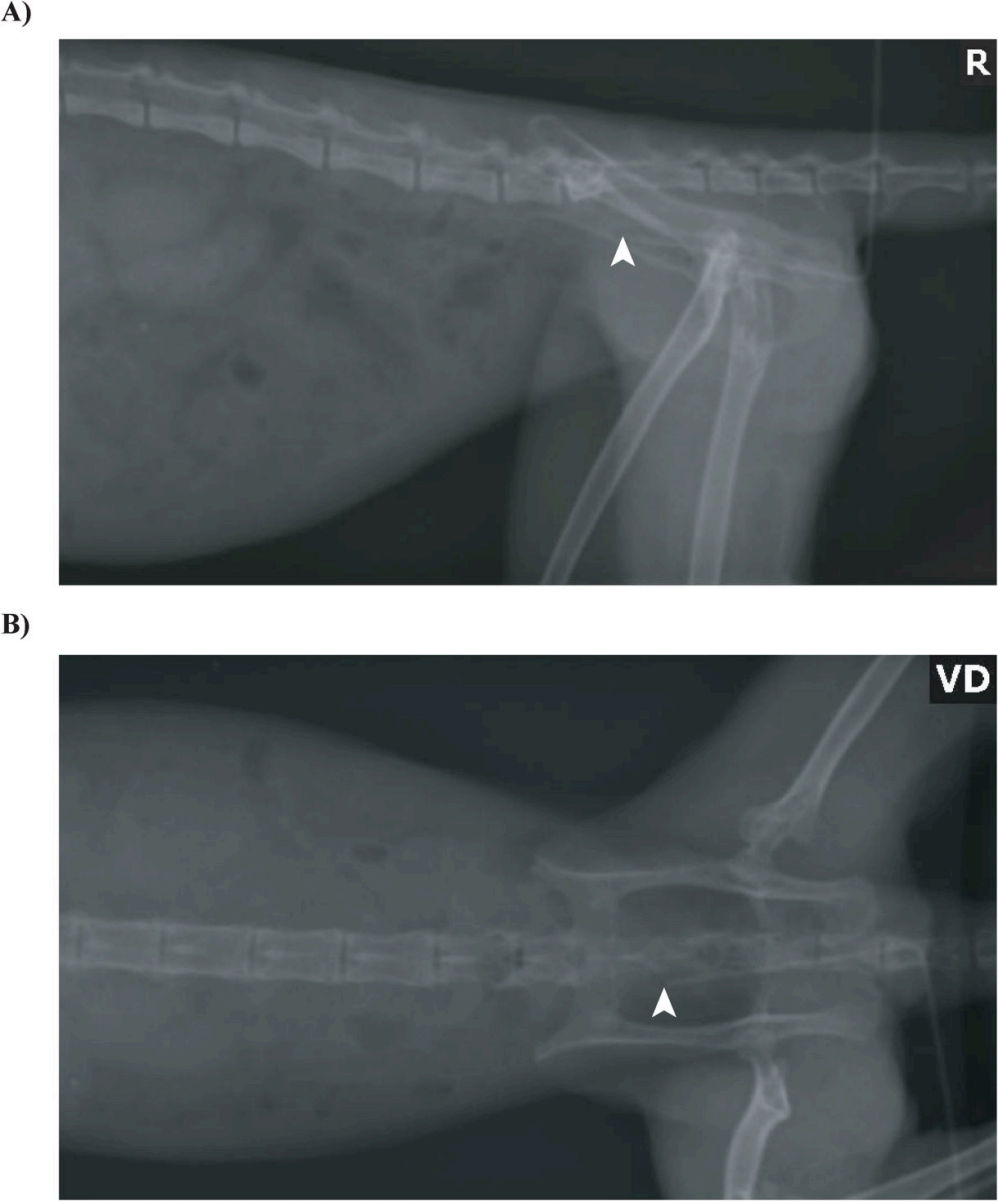
Location of rectal swab sampling. A sedated adult marmoset was radiographed (Toshiba Rotanode at 40 kVp and 3.75 mAs) as a routine procedure. A Calgiswab was inserted in the rectum and advanced to 3 cm past the anal verge. This swab was bent 90° at the 3 cm mark on the shaft so that rest of the shaft could be placed flush with the animal’s skin to indicate the entrance of the rectum. A white arrowhead marks the tip of the swab in the right lateral radiograph (A) and ventrodorsal radiograph (B).

To standardize rectal sample collections, all rectal swabs were demarcated at 3 cm from the tip of the swab with a small kink in the metal shaft generated by bending the metal shaft 90° and then bending it back to the original position. The swab was then inserted into the rectum and advanced to the demarcation. Thus, each rectal swab collected a sample in the distal colon at 3 cm from the anal verge. A radiograph of a sedated marmoset with a swab inserted into the rectum 3 cm past the anal verge is shown in Fig 1 to demonstrate sampling location. To ensure correct positioning for the radiograph and aid in visualization of the rectum, the swab was bent 90° at the 3 cm mark so the remainder of the swab shaft could be placed flush to the animal’s skin.

### Microbiome of rectal swabs is comparable to fecal microbiome profile

The primary goal of this study was to determine differences between the microbiome of a fecal sample compared to a rectal swab sample from the same animal at the same time of collection. The animals selected for this study were all housed in the same facility and within two distinct animal housing rooms (Table 1). One female marmoset was sampled at two different time points for a total of 24 fecal-rectal pairs. At the time of collection most of the animals were clinically healthy but three animals had clinical signs compatible with GI disease. The characteristics of the rectal swab were noted at the time of collection and a descriptive as well as numerical ranking systems was created to describe the amount of material on the swab (Table 1). Free of visible fecal material (FVFM) indicates there was no visible fecal material on the swab (numerical rank = 0) where as “maximal” indicates the swabs that had the most fecal material on the swab (numerical rank = 4). Following DNA extraction, DNA concentrations ranged from 0.62–37.5 ng/μl in the rectal swab samples (Table 1). There was no clear correlation between samples with low DNA concentration and rectal swab ranking, as the five samples with concentrations < 3 ng/μl were classified as FVFM (n = 1), minimal (n = 2) and moderate (n = 2). However, the rectal swabs with highest DNA concentrations were all considered maximal.

**Table 1:** Cohort metadata.

Study ID	Sex	Age (months)	Room	Health Status	Rectal Swab Description	Rectal Swab Numerical Rank	DNA concentration	Reads
1a	Female	12	A	Healthy	Moderate	2	1.3	32,316
1b	Female	13	A	Healthy	Maximal	4	6.59	27,570
2	Female	78	A	Healthy	Moderate	2	8.1	20,607
3	Female	9	B	Healthy	Maximal	4	16.2	16,228
4	Female	70	B	Healthy	Maximal	4	24.8	17,345
5	Female	104	B	Healthy	Minimal	1	1.3	42,350
6	Female	8	A	Healthy	Maximal	4	10.4	18,607
7	Female	7	A	Healthy	Maximal	4	13.8	33,659
8	Female	28	B	GI Disease	Minimal	1	2.58	22,150
9	Female	9	B	Healthy	Considerable	3	8.15	25,496
10	Female	31	A	GI Disease	FVFM	0	10.2	20,564
11	Female	16	A	GI Disease	FVFM	0	4.6	23,679
12	Male	19	B	Healthy	Maximal	4	9.98	23,808
13	Male	6	B	Healthy	Maximal	4	20.9	20,855
14	Male	19	B	Healthy	Maximal	4	37.5	29,680
15	Male	62	B	Healthy	Moderate	2	2.3	34,603
16	Male	19	A	Healthy	FVFM	0	0.62	12,288
17	Male	24	A	Healthy	Maximal	4	22.3	16,989
18	Male	16	A	Healthy	FVFM	0	3.21	33,029
19	Male	32	B	Healthy	FVFM	0	4	34,576
20	Male	69	B	Healthy	Maximal	4	28.4	13,614
21	Male	23	B	Healthy	Considerable	3	12.3	35,157
22	Male^a^	80	A	Healthy	Maximal	4	12.2	20,387
23	Male^a^	81	A	Healthy	Moderate	2	9.38	19,778

Metadata for each animal included in this study. FVFM; Free of visible fecal material

^a^Designates the two males that were vasectomized.

**Fig 2:**
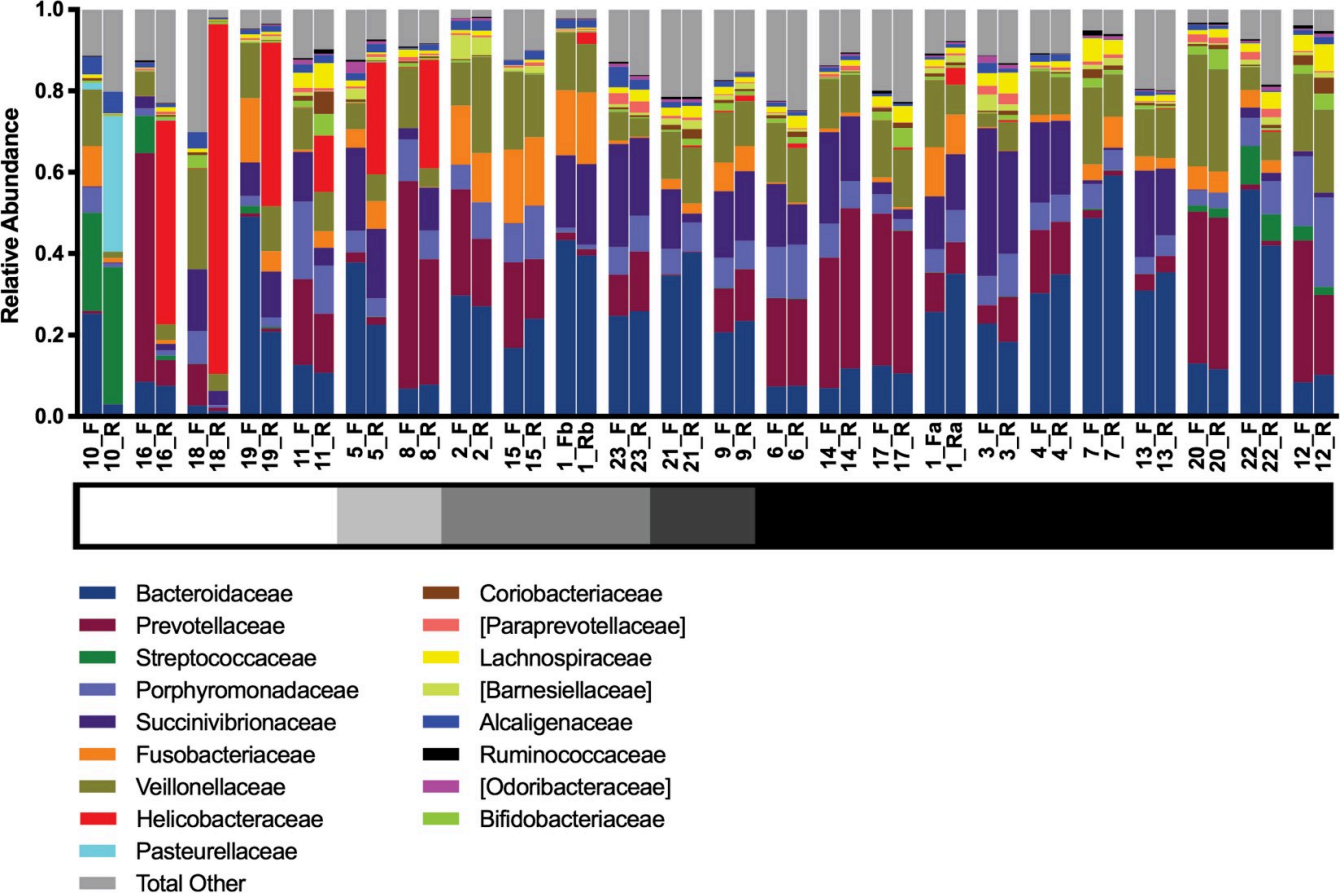
Bacterial community composition of fecal (F) and rectal (R) samples. Each animal in the study is listed by study ID listed in Table 1. The relative abundances of sequences classified to the order level with abundances <1% omitted for convenience. Animal #1 in this study was sampled twice 27 days apart. Thus, the samples are designated as 1_Fa and 1_Ra and 1_Fb and 1_Rb. A greyscale bar under the study ID indicates the relative amount of material on the rectal swab (white = FVFM [0] and black = maximal [4]).

**Fig 3:**
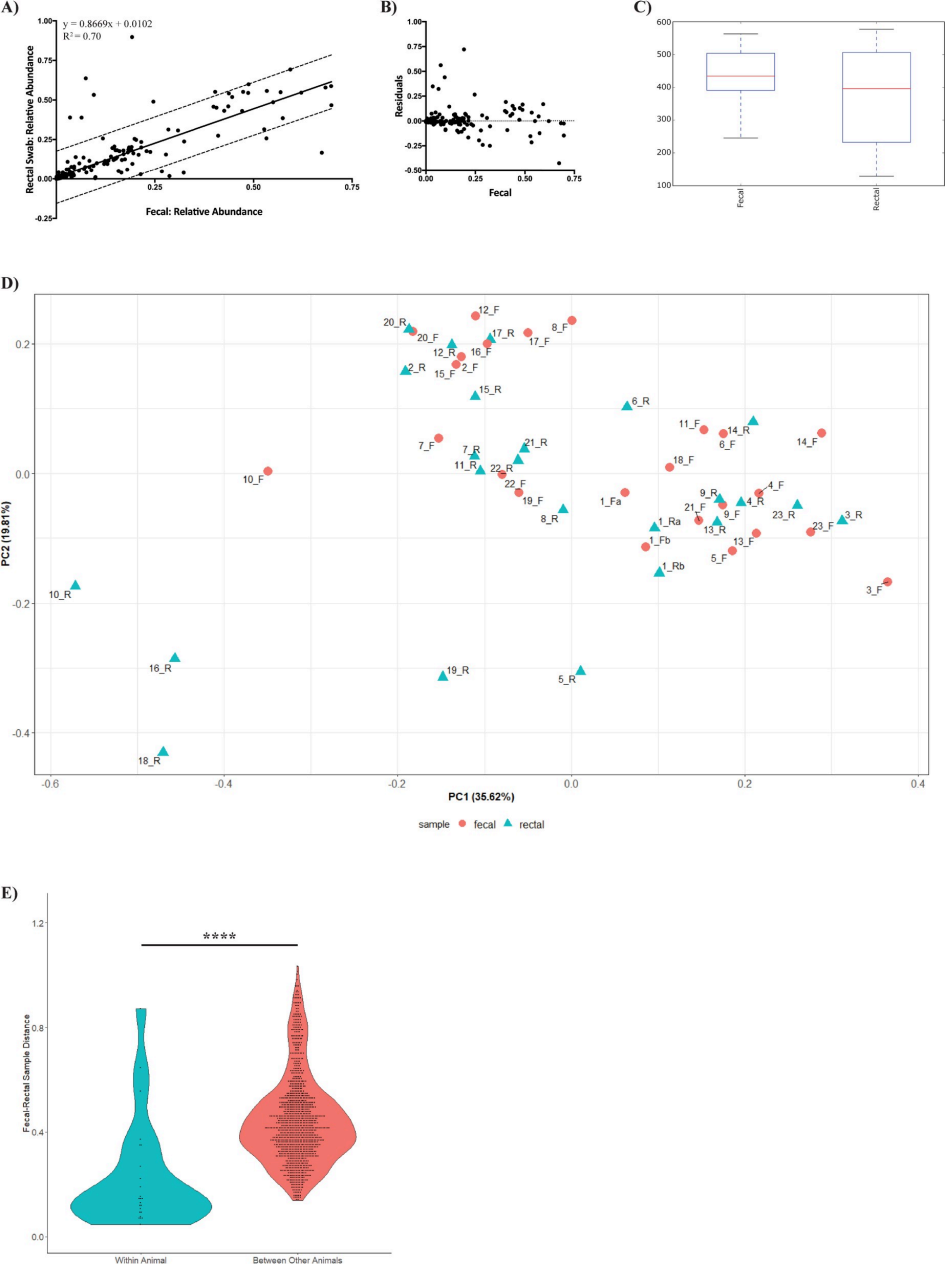
Microbiome analysis of all fecal-rectal pairs collected in this study. A) Relative abundance at the phyla level of each rectal swab sample was plotted against its paired fecal sample. A linear regression model was used to fit the data with a r^2^ value of 0.70 and a standard error of regression (S) of 0.08 to demonstrate how comparable the relative abundance of each rectal swab is to its paired fecal sample. B) A residuals plot of the linear regression fit from A. The residual plot shows a random arrangement of residuals, however there are some residuals that stand out. C) Boxplots showing the α diversity metrics calculated by using the chao1 index in QIIME. No significant difference observed between the fecal and rectal samples. D) Principal Coordinates Analysis (PCoA) of the weighted Unifrac distance representing the β diversity between all the samples E) Distance plots based on the weighted Unifrac distance of fecal and rectal swab samples according to group. Distances between fecal and rectal samples are lower within a marmoset compared to all other marmosets in the study (*P* value: <0.0001).

To determine if the microbiome between a fecal sample and rectal swab sample from the same animal were comparable, 16S ribosomal RNA gene sequences were analyzed for each sample. Due to normalization of samples prior to sequencing, the number of reads obtained for analyzing rectal swab samples ranged from 12,288 to 42,350 reads. No discernible pattern was observed between DNA concentration and read coverage, and the number of reads were deemed sufficient for further analysis. Analysis of the bacterial community composition of each sample demonstrated similar microbial profiles between most paired samples (Fig 2). Overall the fecal-rectal samples are dominated by the phylum *Bacteroidetes*, composed mainly of the families *Bacteroideceae* and *Prevotellaceae*. The phyla *Firmicutes* and *Proteobacteria* share similar relative abundances in this cohort. The families *Veillonellaceae* and *Succinivibrionaceae* were the dominant representatives *Firmicutes* and *Proteobacteria*, respectively. However, the bacterial community of seven fecal-rectal pairs appeared discordant (study IDs: 5, 8, 10, 11, 16, 18, and 19) (Fig 2). Interestingly, all of the discordant pairs include rectal swabs classified as no visible fecal matter or minimal. The ranking scale to portray the relative amount of material on the rectal swab at time of collection is shown in greyscale below the study IDs in Fig 2. To determine if the relative abundance at the phylum level between each pair were comparable, the relative abundance of each phylum from a fecal sample was plotted against the relative abundance of the phylum from its corresponding rectal swab sample. A linear regression model was used to fit the data with a r^2^ value of 0.70 and a standard error of regression (S) of 0.08 (Fig 3A). A residual plot of this fit confirms that the linear model is appropriate for this data (Fig 3B). We next determined if there was a significant difference in diversity metrics between a rectal swab and fecal sample. α diversity metrics calculated by using the Chao1 index showed no significant differences in the richness in bacterial diversity between fecal samples and rectal samples (P = 0.103, Fig 3C). Principal Coordinates Analysis (PCoA) was used to visualize the differences in β diversity between all the samples using the weighted UniFrac distance (Fig 3D). When the data was classified by collection site, no significant differences or sample clustering were observed indicating both sites had a similar abundance and configuration of the microbiome based on sampling site (P = 1.0). Next, we evaluated the similarities of the bacterial communities by calculating a distance matrix and grouping a) distances within an animal’s fecal-rectal pair, and b) between a rectal or fecal sample from an individual compared to a sample from another marmoset. The average distance between fecal-rectal pairs (mean, 0.2257) was significantly different from the average distance between an individual’s sample and a sample from another marmoset (mean, 0.4423; P-value <0.0001), implying that sites within an animal are more closely related than the same site between animals. (Fig 3E).

### Exclusion of rectal swabs with no visible fecal matter improves correlation with fecal samples

**Fig 4:**
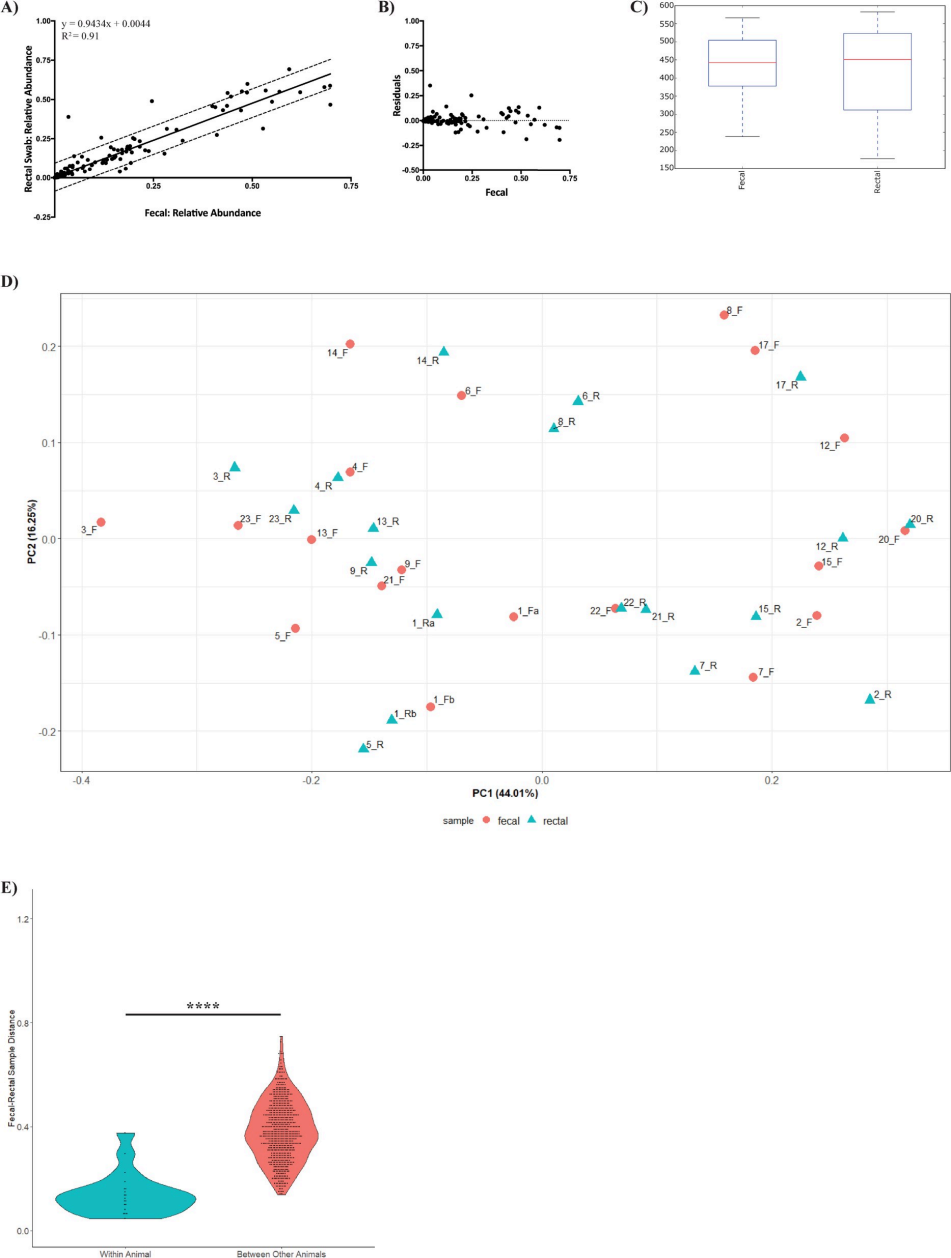
Microbiome re-analysis of all fecal-rectal pairs collected in this study. Re-analysis of fecal-rectal pairs with the omission of 5 pairs that were noted to have FVFM rectal sample during sample collection. A) Relative abundance at the phyla level of each rectal swab sample was plotted against its paired fecal sample. A linear regression model was used to fit the data with a r^2^ value of 0.91 and a standard error of regression (S) of 0.05 to demonstrate how comparable the relative abundance of each rectal swab is to its paired fecal sample. B) A residuals plot of the linear regression fit from A. The residual plot illustrates a random arrangement of residuals with no clear outliers. C) Boxplots showing the α diversity metrics calculated by using the chao1 index in QIIME. No significant difference observed between the fecal and rectal samples. D) Principal Coordinates Analysis (PCoA) of the weighted Unifrac distance representing the β diversity between fecal and rectal swab samples with visible fecal contents. E) Distance plots based on the weighted Unifrac distance of fecal and rectal swab samples according to group. Distances between fecal and rectal samples are lower within a marmoset compared to all other marmosets in the study (*P* value: <0.0001).

**Fig 5:**
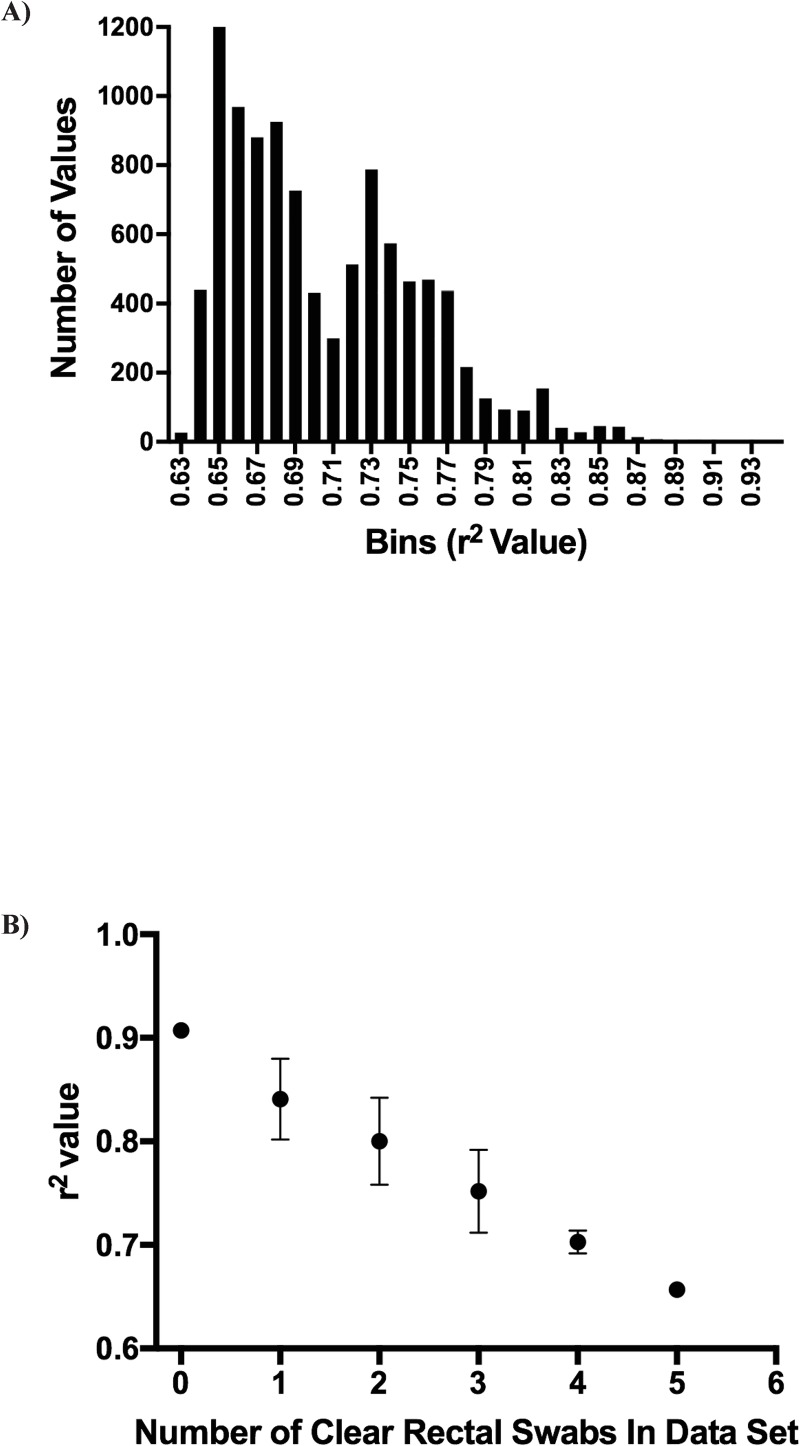
Computer simulated confirm significance of FVFM. A) r^2^ values were simulated by omitting 10,000 combinations of five random samples from the dataset. B) A plot of r^2^ values vs the number of FVFM rectal swabs included in the data set reveals that the r^2^ values increased and were optimized with increased removal of FVFM samples.

Analysis of the relative abundance at the family level revealed that fecal-rectal pairs from marmosets with study IDs 5, 8, 10, 11, 16, 18, and 19 were discordant. Sample pairs 8, 10, 11, 16, 18 and 19 had distances between fecal and rectal pairs > 0.3 (Fig 3E). Sample pair 5 was categorized as minimal and the distance between the paired samples was 0.27 which was closer to the overall mean. Upon further review of collection records, it was noted that five of the seven rectal swabs (study IDs: 10, 11, 16, 18, and 19) were noted as FVFM during collection. Furthermore, data points with large residuals in the residual plot (Fig 3B) suggest the possibility of outliers in the dataset. Interestingly, two of the other fecal-rectal pairs observed to be discordant in the relative abundance analysis (Fig 2), were recorded to be the only rectal swabs with minimal material on the swab. The data were reanalyzed based on the clinical description of the rectal swabs and paired samples with a rectal swab marked as FVFM during collection were omitted. Other rectal swabs described as having a minimal material were included in the re-analysis of the data. FVFM is an objective and easily defined criteria and that is the reason only those rectal swabs were omitted. Re-analysis with those 5 pairs omitted resulted in a better fit of the data as evidenced by a r^2^ value of 0.91 with S of 0.05 (Fig 4A). The residual plot for this new linear regression fit has overall smaller residuals and lacks clear outliers (Fig 4B). No significant differences by sampling site were observed in either alpha (P = 0.39) or beta diversity (P = 1.0) indicating that the microbiome profiles could not be separated as having a fecal or rectal signature (Fig 4C and 4D). Furthermore, the comparison of distances within a sample a pair (mean, 0.1424) and between animals (mean, 0.3834; P-value<0.0001) was still significantly different, showing no negative effect from the omission of the five outliers (Fig 4E). To ensure that the FVFM rectal samples were responsible for the discordant pairs, a computer simulation omitting 5 of 24 paired samples at random was iterated 10,000 times and revealed that the r^2^ value only improved with omitting the 5 FVFM paired samples (Fig 5).

## Discussion

Fecal samples are used as the representative sample in the majority of studies analyzing the gut microbiota of humans and animal models[11,17,18]. The aim of this study was to determine if a rectal swab can be used as a proxy for a fecal sample by testing if the microbiome profiles from a rectal swab and a fecal sample from the same marmoset are comparable. Comparing the relative abundance at the phylum level of each fecal-rectal paired demonstrated that the data fit a linear model with a r^2^ value of 0.91 with S of 0.05 indicating that the relative abundance was in good agreement between the two sample types. There were no differences in the diversity metrics between rectal and fecal samples and the weighted UniFrac distance analysis showed significantly lower distances between fecal-rectal pairs of the same animal than distances between an individual and another marmoset. These findings indicate that rectal swab samples are comparable to fecal samples for microbiome analysis using 16S ribosomal RNA gene sequences.

The previous microbiome study in marmosets used rectal swab sampling and showed that the predominant genera were *Fusobacterium B* and *Bifidobacterium* in the phyla *Fusobacteria* and *Actinobacteria* respectively. The overall abundances of *Fusobacterium B* and *Bifidobacterium* however differed depending on whether the animals were housed in a conventional colony or a SPF barrier colony[10]. In our study, the microbiome profiles of the fecal-rectal paired samples were dominated by the phylum *Bacteroidetes*. The difference in the microbiome profiles observed between the two studies could be due to genetic differences, diet, or other environmental differences between the colonies. As more microbiome studies are published from different colonies it will be crucial to compare the experimental methods utilized as well as husbandry parameters and clinical history of the marmosets in each study.

An important consideration in this study was the removal of 5 fecal-rectal pairs study IDs: 10, 11, 16,18,19) in the final analysis. During physical examination, the veterinarian noted 5 rectal swabs as FVFM at the time of collection. Initial analysis with these 5 pairs resulted in a linear regression model with a r^2^ value of 0.70 and a standard error of regression (S) of 0.08. We hypothesized that these samples were not sampling the fecal contents. Once these 5 fecal-rectal pairs were removed and the data re-analyzed, the linear regression model improved to r^2^ value of 0.91 from 0.70. The FVFM swabs may not have enough luminal content material on the swab but instead may have content from the mucosa. Consistent with this argument is the observation that 4 out of 5 of these FVFM samples have an increase abundance of the order *Campylobacterales* including the family *Helicobacteraceae* compared to the other rectal samples. Indeed, members of this family are known to be inhabitants of the mucus of the intestinal crypts and have been shown to be inhabitants of the marmoset lower bowel and have been detected in feces[19,20]. Furthermore, Yasuda et al. demonstrated in rhesus macaques that the *Helicobacteraceae* family is heavily enriched in the microbiome profiles from the distal colon mucosa compared to the distal colon lumen contents and feces[16]. Based on the current study, rectal swabs with moderate to maximal amounts of fecal material are suitable proxies for fecal or luminal samples from the lower bowel in marmosets. Standardizing collection techniques will lead to improved reproducibility and the ability to compare data across marmoset populations both in the wild or in captivity, as fecal samples are frequently used due to ease of sampling in microbiome studies. However, we recognize that the microbiome adjacent to the host GI mucosa is markedly different as exemplified by the FVFM samples. The microbiota closely associated to the gut has important and not well-understood biological functions, such as immunomodulation[21], but is often masked by the more abundant fecal and luminal bacteria. Unfortunately, the methods presented cannot consistently produce samples to survey the mucosa-associated microbiome.

While the focus of this study was to compare different sample types collected from the same animal, several clinical observations from the cohort will require further studies to determine their significance to marmoset biology. At the time of sample collection, 87% of the animals were clinically healthy while 3 of the 23 animals had clinical signs compatible with GI disease. Interestingly, 2 of these 3 animals had FVFM rectal swabs and the other animal had a rectal swab with minimal material. These findings suggest that obtaining rectal swabs with fecal material in animals with clinical signs of GI disease may be challenging. Further standardized studies with a larger number of animals are necessary to establish what the microbiome of the colonic mucosa should resemble in health and disease. FVFM or minimal rectal swabs may indeed represent a distinct sample site, namely the colonic mucosa, with a different microbiota than feces, the focus of the current study. Previous studies conducted in humans and other lab animal species have documented correlations between health, disease, medications, and diet[22–24]. While this study did not take into account factors, such as medications, that may modulate the microbiome, ongoing longitudinal studies within our colony aim to determine whether modulation of the microbiome due to disease or treatment may be predictive of future development of GI disease. In the current study, a single marmoset was sampled twice over the course of a month and exhibited a stable colonic microbiota, but a larger cohort is necessary to understand microbial dynamics over longer intervals of time. Furthermore, marmoset microbiome studies at multiple institutions will be informative as inter-institutional variations in diet, husbandry, clinical disease and their management of disease may ultimately affect bacterial composition of the gut microbiome and play a role in differences in prevalence of GI disease and average lifespan of captive marmosets.

This study demonstrated that rectal swabs containing visible fecal content are a reliable method of fecal sample collection and can serve as a proxy of feces in microbiome studies in marmosets. This study highlights the importance of standardized sample collection methods and exclusion of inappropriate samples depending on the design of the experiment. This study also serves as a framework for other institutions and illustrates that a simple and convenient rectal swab sample for lower bowel microbiome analysis can be reliably used. Microbiome studies of different captive colonies will undoubtedly increase our understanding of differences in disease incidence and lifespan. Furthermore, experimental common marmoset models will benefit from understanding the marmoset gut microbiome since the microbiome in other lab animal species is considered an important variable in animal experiments[25].

## Materials and methods

### Animals

Twenty-three common marmosets were used in this study. Seven captive-bred animals were originally sourced from the New England Primate Research Center (NEPRC) and 16 animals were bred at MIT from NEPRC sourced animals. The animals were housed in the same AAALAC International-accredited facility. The cohort of animals in this study were 12 females and 11 males (2 of the males were vasectomized) ranging in age from 6 months to 9 years of age (Table 1). All of the marmosets were group housed except one male marmoset who was temporarily singly housed. All the marmosets were on an animal use protocol approved by the Massachusetts Institute of Technology’s Institutional Animal Care and Use Committee (IACUC) known as the Committee on Animal Care. All procedures were carried out in accordance with National Institute of Health’s guidelines including the Guide for the Care and Use of Laboratory Animals. Custom-designed, stainless steel and polycarbonate cages (30" W x 32" D x 67" H) were used for the housing. The holding room was maintained at 74.0 +/- 2.0° F with a relative humidity of 30% to 70% and a 12:12 h light:dark cycle. Enclosure enrichment was comprised of perches, nest boxes, hammocks, manzanita wood branches, and hanging toys. Foraging trays and acacia gum treats were provided weekly.

The marmoset colony received unrestricted access to water via an automatic watering system plus 2 polycarbonate, 500-mL water bottles per cage. Water bottles and cages were changed every 2 weeks. The main diet fed once a day, consists of biscuits (Teklad New World Primate Diet 8794, Envigo, Madison, WI), briefly soaked in water, supplemented with fruits, vegetables, and additional protein sources including hard-boiled eggs, cottage cheese or ZuPreem (Premium Nutritional Products, Inc., Mission, KS). Marmoset colony health monitoring consisted of semiannual sampling for potentially pathogenic bacteria (including *Salmonella* spp., *Shigella* spp., beta-hemolytic *E*. *coli*, *Klebsiella* spp., and *Campylobacter* spp.) and parasites (including *Enterobius* spp., *Entamoeba* spp., *Giardia* spp., and *Cryptosporidium* spp.). Animals are seronegative for squirrel monkey cytomegalovirus, *Saimiriine herpesvirus 1*, *Saimiriine herpesvirus 2*, and measles virus. Physical examinations are performed at least twice a year on all marmosets over 6 months of age, while complete blood counts and serum chemistries are performed annually and more often as needed.

### Sample collection and storage

All samples were collected by the same veterinarian as part of routine sedated physical exams. All animals were sedated with 6 mg/kg alfaxalone (Alfaxan, Jurox, Kansas City MO) given via intramuscular injection. The animals were fasted for 2–5 hours prior to the sedated physical exam. Calgiswab^®^ Mini Calcium Alginate Tipped Applicators (Puritan Medical Products, Guilford, ME) were used for collecting the fecal sample from the transport carrier as well as for the rectal swab sample. Each animal was transported from their home cage in a clean transport carrier to the exam room. If a transport carrier needed to be re-used for another animal, the carrier was disinfected with Rescue^®^ spray or wipes (Virox Animal Health, ON, Canada) and remained wet for at least 1 minute per the manufacture’s protocol. This study only includes animals in which fecal and rectal swab paired samples were collected at the same time. The fecal sample was collected from the carrier after the animal defecated during the transport to the exam room. A sterile swab was used to collect the fecal sample from the clean carrier after the animal was removed. Sterile swabs were pre-marked at 3 cm from the swab tip with a small bend in the metal shaft. The swab was then inserted into the rectum 3 cm past the anal verge and gently rotated 720°. The characteristics of the feces and/or swab were recorded at the time of sample collection. The swab was inserted into a labeled 2 mL cryotube, and sterile scissors were used to cut the shaft of the swab. The cryotube was then immediately frozen in liquid nitrogen and stored at -80°C prior to use.

### Preparation of DNA from rectal and fecal samples

Swabs and feces were retrieved from -80°C and DNA was isolated using the DNeasy PowerLyzer PowerSoil Kit according to the supplier’s protocol (Qiagen Inc., Valencia, CA). Bead beating of rectal swab and fecal samples was performed with a Next Advance Bullet blender (Troy, NY) for 8 min at the maximum speed. Purity of the prepared DNA was analyzed using Nanodrop 2000C (Thermo Scientific, Wilmington, DE).

### 16S rRNA gene sequencing and analysis

DNA extracted from both rectal and fecal samples was amplified using universal primers of F515 (GTGYCAGCMGCCGCGGTAA) and R926 (CCGYCAATTYMTTTRAGTTT) to target the V4 and V5 regions of bacterial 16S rRNA fused to Illumina adaptors and barcode sequences as described previously[26]. Individual samples were barcoded and pooled to construct the sequencing library, followed by sequencing with an Illumina MiSeq instrument to generate pair-ended 250 × 250 reads. Overlapping pair-end reads were aligned using PEAR[27]. Paired reads shorter than 200 bp were filtered out. Subsequent analysis and normalization were performed using QIIME 1.9.1[28] within the MicrobiomeHelper v. 2.0.0 virtual box[26] Chimeric sequences were removed by comparison to the database Bacteria_RDP_trainset15_092015.fa. Operational taxonomic units (OTUs) present at less than 225 reads, corresponding to 1% of the average number of reads, were removed. The average number of reads for the rectal and fecal swab samples were 24805 and 20479 reads, respectively. Microbial communities were compared by using UniFrac. Sequences were grouped into OTUs at 97% sequence similarity using uclust. Taxonomy was assigned using Ribosomal Database Project (RDP) classifier against GreenGenes database, and sequences were aligned, and phylogenetic tree was built from reference sequences using FastTree. An OTU table showing counts of each OTU in each sample was produced. To control for differences in sequencing depth, OTU tables were rarified at a single sequencing depth[29,30]. Relative abundance of bacterial taxa of each fecal-rectal pair generate in QIIME was analyzed and data fitted using PRISM 7 (GraphPad, La Jolla, CA). Alpha diversity was determined using the Chao1 index. Significant differences in alpha diversity were evaluated using a non-parametric two-sample t-test with p-values < 0.05 considered significant. Beta diversity was determined using weighted UniFrac[31] and the results presented as principal coordinate analysis (PCoA) plots. The QIIME script make_distance_boxplots.py, PRISM 7, Microsoft Excel, R (version 3.4.1 at http://www.R-project.org/) and the ggplot2 (2.2.1) library were used to perform statistical analyses and graphically represent data of the weighted UniFrac distance matrices[32]. Two-sample unequal variances *t*-tests were performed on the distance matrices with P-values < 0.05 considered significant.
